# Nano-TiO_2_ Coating Layers with Improved Anticorrosive Properties by Aerosol Flame Synthesis and Thermophoretic Deposition on Aluminium Surfaces

**DOI:** 10.3390/ma14112918

**Published:** 2021-05-28

**Authors:** Gianluigi De Falco, Giuseppe De Filippis, Carmela Scudieri, Luca Vitale, Mario Commodo, Patrizia Minutolo, Andrea D’Anna, Paolo Ciambelli

**Affiliations:** 1 Dipartimento di Ingegneria Chimica, dei Materiali e della Produzione Industriale, Università degli Studi di Napoli Federico II, P.le Tecchio 80, 80125 Napoli, Italy; gianluigi.defalco@unina.it; 2 Narrando Srl, Via Giovanni Paolo II, 132, 84084 Fisciano, Italy; pe.defilippis95@gmail.com (G.D.F.); cscudieri@unisa.it (C.S.); Luca94univ@gmail.com (L.V.); pciambelli@unisa.it (P.C.); 3 Istituto di Scienze e Tecnologie per l’Energia e la Mobilità Sostenibili, STEMS-CNR, P.le Tecchio 80, 80125 Napoli, Italy; commodo@irc.cnr.it (M.C.); minutolo@irc.cnr.it (P.M.)

**Keywords:** thermophoretic deposition, nanostructured layers, Nano-TiO_2_, anticorrosive coatings

## Abstract

TiO_2_ in the form of nanoparticles is characterized by high photocatalytic activity and high resistance to oxidation, making it an excellent candidate to realize coatings for improving the corrosion resistance of aluminium surfaces. Different coating technologies have been proposed over the years, which often involve the use of toxic compounds and very high temperatures. In this work, an alternative and novel one-step method for the coating of aluminium alloy surfaces with titania nanoparticles is presented. The method is based on the combination of aerosol flame synthesis and direct thermophoretic deposition and allows to produce nanostructured thin coating layers of titania with different features. Specifically, 3.5 nm anatase nanoparticles were synthesized and deposited onto aluminium alloy AA2024 samples. The thickness of the coating was changed by modifying the total deposition time. A thermal annealing treatment was developed to improve the adhesion of nano-titania on the substrates, and the morphology and structures of the coatings were characterized using (ultra violet) UV-vis absorption, scanning electron microscopy, transmission electron microscopy and Raman spectroscopy. The corrosion resistance behavior of the coatings was evaluated by means of electrochemical polarization measurements, coupled with a numerical analysis using COMSOL software. Both the experimental and numerical electrochemical polarization curves showed a significant increase in the corrosion potential of coated substrates with respect to the bare aluminium and a decrease in the current density. The coatings obtained with higher deposition time and greater thickness showed the best performances in terms of the resistance of the aluminium surfaces to corrosion.

## 1. Introduction

Aluminium alloys represent today a primary choice for the manufacturing of structural components, due to their peculiar characteristics such as high mechanical properties (specifically, damage tolerance and tensile strength), low specific weight and the ease of manufacturing [1,2]. For the reasons listed above, aluminium is one of the most used materials in automobiles and commercial vehicles, as well as in the aeronautic industry. On the other hand, a key point in designing structural components made of aluminium alloys is the protection from corrosion, since aluminium alloys could be very sensitive to corrosion [3]. To this aim, surface treatments are widely used since they could lead to an improvement of the electrochemical properties of aluminium-made surfaces without significant mechanical performance losses [4]. Coatings made by TiO_2_ nanoparticle are highly attractive due to the excellent chemical stability and low toxicity of titania even in the form of nanoparticles and the high compatibility with aluminium alloys [5,6,7,8,9]. Several techniques are currently used to realize coatings of nanoparticle layers on different surfaces. The traditional technologies used for protective coatings on aluminium surfaces are conversion coating processes and thermal spray processes, which are very effective to produce surface coatings. However, conversion coating processes, such as chromate conversion and anodic oxidation, involve the use of hexavalent chromium compounds, which are very toxic and dangerous for both the environment and human health [10]. Moreover, thermal spray processes, i.e., plasma spray, require very high temperatures, which could be higher than the aging temperature of aluminium alloys, thus leading to the decay of the mechanical properties of the age-hardened aluminium. Other attractive coatings technologies, such as physical and chemical vapor deposition [11], are limited by relatively high complexity and low production efficiency.

Aerosol Flame Synthesis (AFS) furnishes an easy, cost-effective and scalable method to synthesize nano-TiO_2_ with required properties and to effectively deposit particles in one step as nanostructured coatings on metal alloy surfaces [12]. In AFS, a flame is used as a high-temperature reactor for synthesizing both organic and inorganic solid species in the nanoscale [13,14,15]. High temperature and short residence time can be easily achieved in an AFS reactor, which are the optimal reactor conditions for the synthesis of nanomaterials. In addition, AFS offers the possibility to easily obtain nanostructured films and coatings by placing a substrate downstream of the flame synthesis burner, on which nanoparticles are then deposited by thermophoresis [16,17]. Thermophoresis is a force acting on particles suspended in a medium where a thermal gradient exists, which results in a motion of particles from the high-temperature region towards the lower temperature zone. This method was successfully applied to develop systems able to produce nano-TiO_2_ thin films for applications in photo water-splitting, carbon monoxide sensing, dye sensitized solar cells [18,19,20].

In the light of the above, the objective of the present work was the improvement and application of a previously developed cheap and single-step procedure with few waste and by-products generation for the coating of aluminium alloy surfaces with TiO_2_ nanoparticles. The coating technology is proposed as an alternative to the traditional technologies currently used to realize titania nanocoatings on a metal surface. An experimental and numerical characterization of the improved electrochemical behavior of coated aluminium surfaces was performed to investigate the anticorrosive properties of nano-TiO_2_ layers. 

## 2. Materials and Methods

TiO_2_ nanocoatings were produced via flame synthesis and direct thermophoretic deposition on cold substrates. The flame synthesis apparatus consists of a custom-made honeycomb burner and a high-pressure syringe pump to feed the precursor solution. Thermophoretic deposition of the flame-formed nano-TiO_2_ particles is based on a rotating disk system that allows metal substrates to be inserted in the flame by a fast insertion procedure that maintains the substrate at room temperature. More details of the experimental system and a sketch of the experimental setup can be found in previous works [8,21]. The rotating disk allows aluminium AA2024 substrates to be allocated on its surface; either small substrates (diameter = 1.6 cm, thickness = 3 mm, total coating area of 2 cm^2^) and a larger single annulus-shaped substrate (outer diameter OD = 25.6 cm, inner diameter ID = 22.4 cm, thickness = 6 mm, total coating area of 120 cm^2^) can be allocated over the rotating disk to be coated. The weight percentage compositions of other metals in AA2024 aluminum alloy substrate are: Cu 3.8%–4.9%, Cr < 0.1%, Fe < 0.5%, Mg 1.2%–1.8%, Mn 0.3%–0.9%, Si < 0.5%, Zn < 0.25%, Ti < 0.15%.

The custom-made flame reactor used for the synthesis of nano-TiO_2_ consists of a premixed laminar flame of ethylene and air, with a cold gas velocity *v*_cg_ = 95 cm/s and a flame equivalent ratio Φ = 0.95. The flame is doped with a 0.5 M solution of titanium tetraisopropoxide (TTIP) (Sigma Aldrich, Saint Louis, MO, USA) into ethanol, fed to the reactor at a flow rate of 900 μL/min. The AFS reactor in those operating conditions produces pure anatase TiO_2_ nanoparticles with dimensions of about 3 nm [21]. TiO_2_ nanostructured coatings with different thicknesses were obtained, operating the rotating disk with a rotational speed of 600 rpm and changing the total deposition time t_d_ from 15 s up to 80 s. In order to increase the amount of sample surface protected by nano-titania, the coatings procedure was also performed on both surfaces of selected samples.

After the deposition process, all the coated samples underwent an annealing process performed by putting the coated substrate in direct contact with the hot plate surface. The hot plate was placed inside a chemical hood under controlled atmosphere conditions. The hot plate annealing technique reduces the fluctuation of the annealing temperature with respect to an oven, where the heat transfer by convection usually has an error of ±15 °C [22].

Characterization of nanocoatings was performed by using different experimental techniques. Transmission Electron Miscroscopy (TEM) images were acquired on an HRTEM FEI Tecnai (FEI, Hillsboro, OR, USA) electron microscope, operating at 200 kV with a LaB6 filament as the source of electrons and equipped with an energy-dispersive X-ray spectroscopy (EDX) probe. For the preparation of the TEM sample, TiO_2_ powders were mechanically removed from the coated samples and suspended in ethanol. TiO_2_ nanoparticles were then deposited dropwise on carbon-coated electron microscope grids. Scanning electron microscopy (SEM) images were obtained with an LEO 1525 (One Zeiss Drive, Thornwood, NY, USA) microscope. X-ray diffraction (XRD) spectra were measured with the aim of a Bruker D8 Advance X-ray diffractometer using CuKα radiation.(ultra violet) UV-vis absorption spectra were acquired on nanoparticle coatings deposited on quartz substrates by means of an Agilent UV-visible 8453 (Agilent, Santa Clasa, CA, USA) spectrophotometer and on NaCl solutions after the corrosion tests using a UV-visible Thermo-Fisher Evolution 60S (Thermo Fisher Scientific, US) spectrophotometer. An annealing procedure, when required, was performed using a Stuart undergrad stirrer hotplate model US152 (Stuart, Stone, UK).

**Figure 1:**
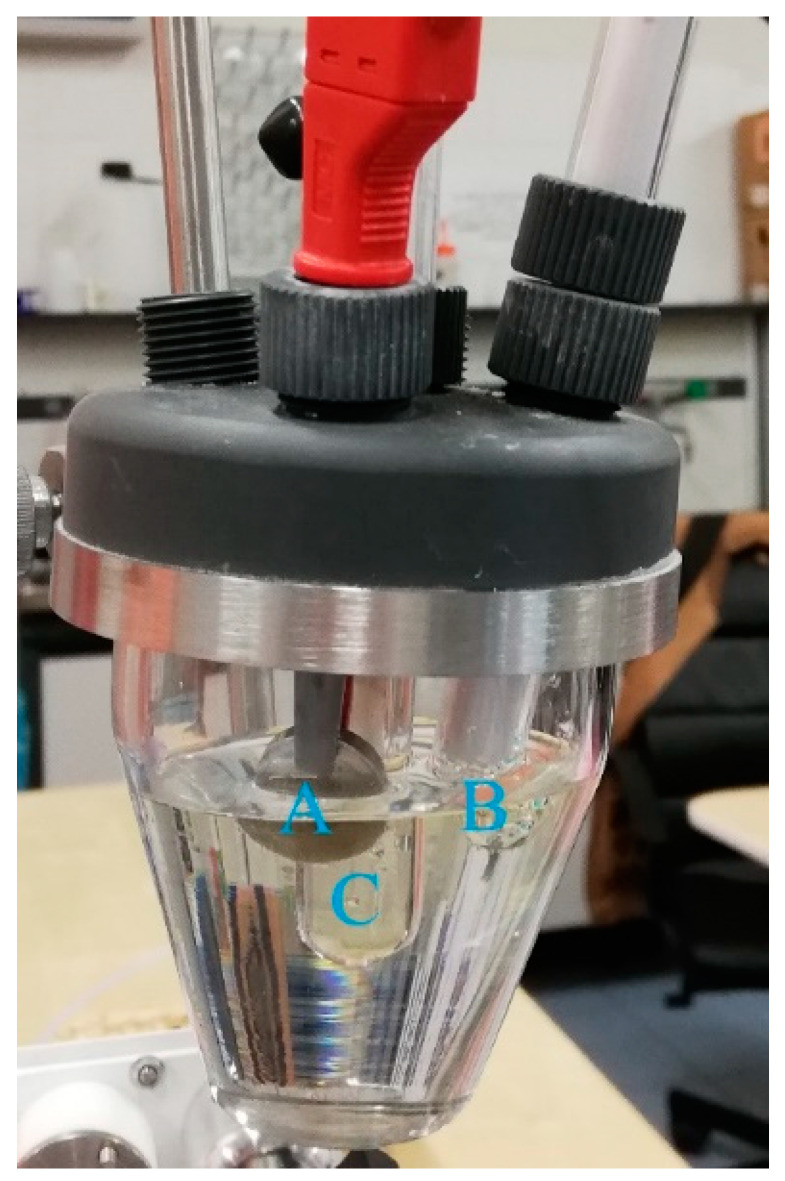
Picture of the electrochemical cells used for corrosion tests.

The corrosion resistance of uncoated and TiO_2_-coated circular aluminium AA2024 substrates was carried out by performing electrochemical measurements with a PGSTAT302N Autolab (Metrohm AG, Switzerland) Potentiostat/Galvanostat controlled by Nova 2.0 software (Metrohm AG, Switzerland). An electrochemical cell with three standard electrodes (Metrohm AG, Herisau, Switzerland) was used, adopting the following configuration: a calomel electrode, used as a reference electrode (RE), a platinum electrode that acts as a counter electrode (CE) and each sample (with an area of ~2 cm^2^) was connected to a clamp acting as a working electrode (WE). Figure 1 shows a picture of the electrochemical cell used for corrosion tests. It is possible to observe the working electrode (A), that is, the AA2024 alloy disk, the platinum counter electrode (B) and the reference electrode (C).

Measurements were carried out at room temperature in an electrolytic solution of sodium chloride (NaCl), quiescent and aerated (representative of the corrosive environment), and almost neutral (pH 6.5 ± 0.2) at 0.6 M NaCl. All electrochemical tests were performed after 1 h of immersion of the samples at open circuit potential, E_ocp_. Furthermore, the linear anodic polarization tests were conducted at wide ranges of the potentials and at a scan rate of 1 mV·s^−1^. To evaluate the effect of the deposition time of TiO_2_ on the aluminium surface in terms of corrosion resistance, the treatment was carried out at different total deposition time td. Using the Tafel polarization tests, the values of corrosion potential (E_corr_), corrosion current density (I_corr_), corrosion rate and polarization resistance (R_p_) were determined.

A galvanic cell model was developed using the COMSOL Multiphysics simulation software (COMSOL, Version 5.5, 2019) in order to generate and compare results with the experimental measurements of the corrosion tests.

The model was based on the theory of heterogeneous chemical reactions. When a corrosion process occurs, surface reactions involve a reduction reaction and an oxidation reaction, in which a metal structure is in contact with an electrolyte. The circuit is closed through electrochemical reactions and current transport through ion conduction in the electrolyte.

The simulation program was analyzed to understand how the model could be developed as close to the experiments as possible. Moreover, a preliminary literature study was performed to obtain all the input data for the software needed to properly characterize the system, which also affects the nature of the electrodes and the electrolyte. 

During the modeling activities, some issues related to the nature of the AA2024 alloy were faced. AA2024 is mainly composed of aluminium but also other metals; it has microstructural properties, which influence the bulk properties with respect to corrosion and corrosion phenomena. Therefore, a model was created to simulate these processes using the COMSOL Multiphysics software, particularly the anodic polarization, which took into account the two reactions that occur and develop at the anode and cathode. The two reactions refer to aluminium which is the main component of the alloy, and to oxygen responsible for oxidation:

(1) $$Al=Al^{3+}+3e^{-}\;\;\;\;\;\;\;\;E_{\mathrm{eq}}=-1.66\;V$$

(2) $$O_{2}+2H_{2}O+4^{e-}=4OH^{-}\;\;\;\;\;E_{\mathrm{eq}}=+0.81\;V$$

On the other hand, the nano-titanium oxide coatings were treated as a passivating layer.

## 3. Results and Discussion

**Figure 2:**
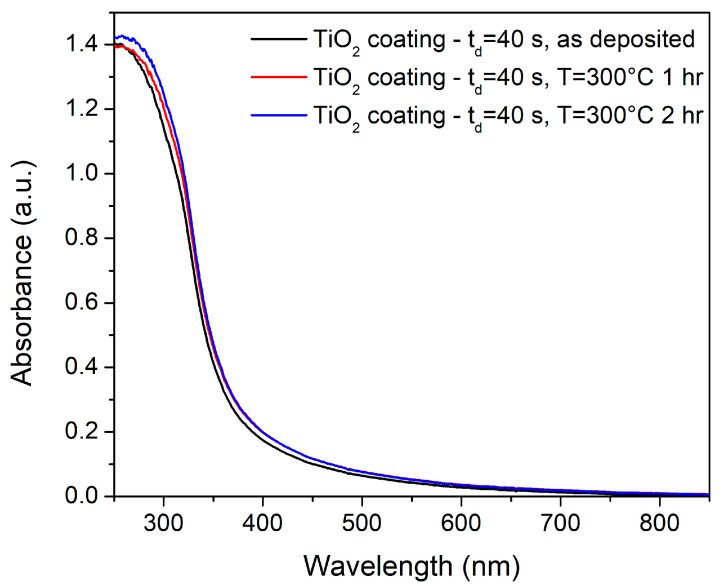
(Ultraviolet) UV-visible absorption spectra acquired on nano-TiO_2_ coatings with t_d_ = 40 s as-deposited, annealed at 300 °C for 1 h and annealed at 300 °C for 2 h.

Figure 2 reports a comparison between UV-visible absorption spectra acquired on nano-TiO_2_ coatings with a deposition time t_d_ = 40 s that underwent different annealing conditions: as-deposited, annealed at 300 °C for 1 h and annealed at 300 °C for 2 h. Annealing of thin films was demonstrated to result in an increase in the film crystallization and densification and so in a better adhesion of the coating to the substrate [23] The annealing parameters were set at 300 °C and 30 min. The parameters were chosen in order to avoid any temperature-induced phase transformation from anatase to rutile, which occurs at around 650–700 °C [24,25]. Anatase is the most photoactive phase of TiO_2_ [26], and so it is the ideal phase for photoactivated processes such as corrosion protection mechanisms. Moreover, previous works found that 800 °C is a critical annealing temperature at which nanoscale structural and optical properties exhibit significant changes [25]. The spectra reported in Figure 2 show a high absorbance in the Ultra-Violet A/Ultra-Violet B region, as well as an absorption tail in the visible region. No significant changes in the absorption maximum and the shape of the curve within the experimental uncertainties can be detected on the annealed samples. Therefore, we can assume that the annealing process performed at 300° C for 60 min resulted in better adhesion of the coatings to the substrates without any modification of the optical and structural properties of the nanocoatings.

**Figure 3:**
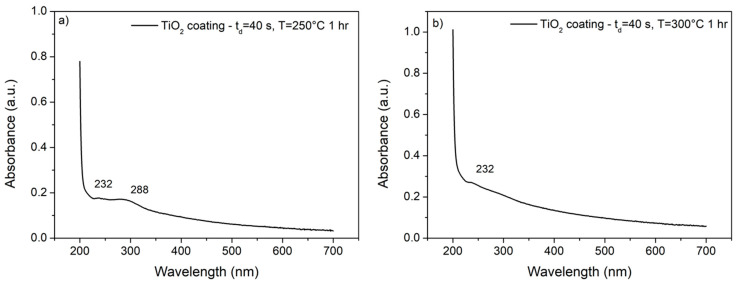
UV-visible absorption spectra of the NaCl solution after the corrosion test on nano-TiO_2_ coated AA2024 substrate with t_d_ = 40 s and annealed at 250 °C for 1 h (**a**) and of the NaCl solution after the corrosion test on nano-TiO_2_ coated AA2024 substrate with t_d_ = 40 s and annealed at 300 °C for 1 h (**b**).

Further characterization of the adhesion of annealed samples on the substrates was performed by measuring UV-vis spectra on electrolytic solutions of sodium chloride used for the corrosion tests. Figure 3 reports the spectrum acquired on the solution used to perform the corrosion test on nano-TiO_2_ coated AA2024 substrate with t_d_ = 40 s and annealed at 250 °C for 1 h, and the spectrum acquired on the solution used to perform the corrosion test on nano-TiO_2_ coated AA2024 substrate with t_d_ = 40 s and annealed at 300 °C for 1 h. It can be noticed that the annealing process performed at 250 °C does not provide sufficient adhesion of the coating since a peak at 288 nm can be detected and attributed to the presence of a small number of titania nanoparticles dissolved in the solution. On the other hand, the annealing process performed at 300 °C resulted in an optimal adhesion of the coating since no peaks related to the presence of TiO_2_ in the solution are detected. The signal at 232 nm is likely due to the presence of aluminum traces in the solution. 

**Figure 4:**
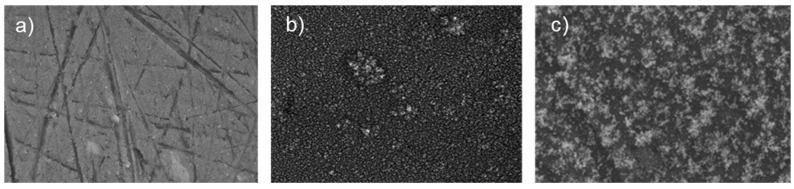
SEM images of bare AA2024 disk (**a**), t_d_ = 40 s nano-TiO_2_ coated AA2024 (**b**) and t_d_ = 80 s nano-TiO_2_ coated AA2024 (**c**).

Figure 4 shows SEM images acquired on the surface of a bare AA2024 disk and AA2024 disks covered by nano-TiO_2_ coating with a deposition time t_d_ = 40 s and t_d_ = 80 s.

The bare sample shows signs on the surface, which are due to the preparation and the finishing processes. On the other hand, the images on both coated samples reveal the presence of a uniform coating of titania nanoparticles on the entire surface. 

**Figure 5:**
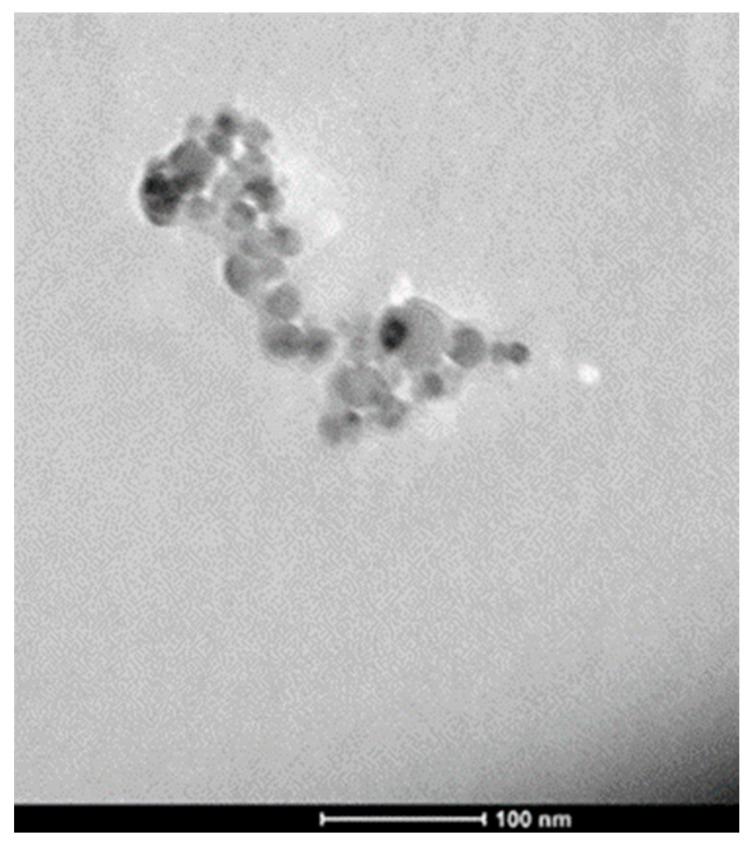
TEM image obtained on an aggregate of TiO_2_ nanoparticles.

The morphology of nanoparticles composing the coating was analyzed using a TEM microscopy. Figure 5 shows a TEM image obtained on an isolated aggregate of TiO_2_ nanoparticles mechanically removed from the t_d_ = 80 s coated aluminium substrate. It is worth noting that the dimension of primary particles in the aggregate is in quite good agreement with the dimensions obtained from previously reported characterizations [21].

**Figure 6:**
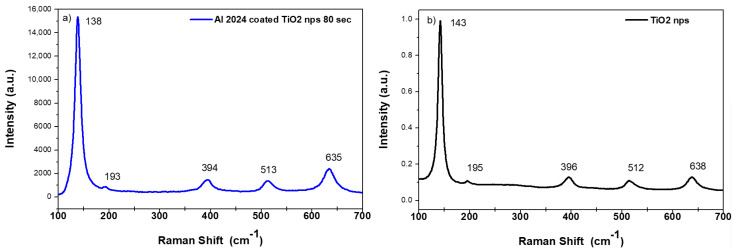
Raman spectrum measured on t_d_ = 80 s coated AA2024 substrate (**a**) and Raman spectrum measured on TiO_2_ nanoparticles removed from the aluminium substrate (**b**).

Raman spectra were measured on t_d_ = 80 s coated aluminium substrate, as well as on powder of TiO_2_ nanoparticles mechanically removed from the aluminium substrate. In both spectra shown in Figure 6, all the peaks related to the crystalline phase of anatase can be clearly detected.

**Figure 7:**
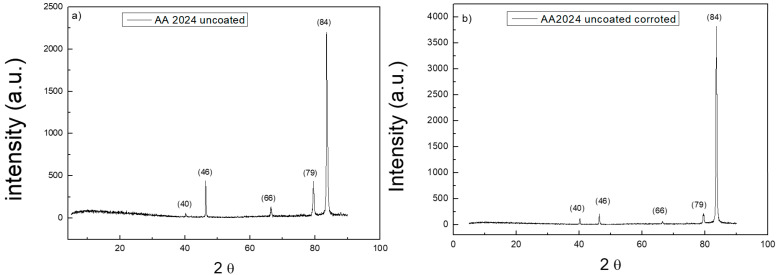
XRD spectra of the bare AA2024 disk before (**a**) and after the corrosion test (**b**).

**Figure 8:**
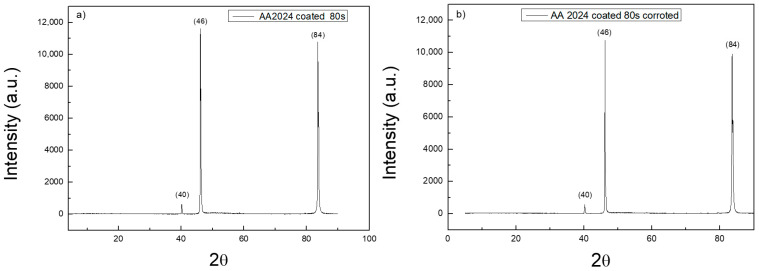
XRD spectra of the t_d_ = 80 s coated AA2024 disk before (**a**) and after the corrosion test (**b**).

X-ray diffraction measurements were also performed on uncoated and coated samples. Figure 7 reports XRD spectra on the surface of bare AA2024 substrate before and after the corrosion tests. Figure 8 reports XRD spectra on the surface of t_d_ = 80 s coated AA2024 substrate before and after the corrosion tests. All the spectra show the typical peaks of aluminium. There is no evidence of signals of the other components of AA2024, nor of the intermetallic phases that are typically formed in the alloy. The spectrum acquired on the substrates after the corrosion test reveals the disappearance of the peaks at 66 and 79°. The presence of TiO_2_ nanoparticles on the coated sample is not detected due to the low mass amount of titania in the micrometric thick layers, which is below the sensitivity of the instrument. The spectra of the coated samples are both very similar to that of the bare sample before testing.

Table 1 shows all the values relating to the XRD peaks. The ratio between the pre-corrosion and post-corrosion normalized intensities of the samples was calculated. In particular, it can be noted that for the uncoated sample, the intensity of the peaks undergoes a considerable variation, while for the coated sample, the intensities remain constant. This is evidence of the protective effect of the titania coating on the whole alloy surface. The peaks of the intermetallic alloys and of titania were not detected, likely due to their very low concentration.

**Figure 9:**
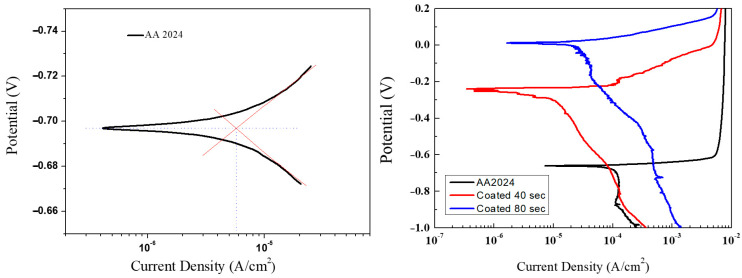
Polarization curves measured on uncoated bare substrate and on samples coated on both sides with t_d_ = 40 s and t_d_ = 80 s.

Several coated substrates were prepared to perform the characterization of the electrochemical performances. AA2024 aluminium disks were coated with TiO_2_ nanocoatings at two different deposition times, namely t_d_ = 40 s and t_d_ = 80 s. For both deposition times, different samples were prepared to perform the deposition procedure on just one side and on both sides of the substrates. The results of the corrosion tests through the linear anodic polarization on the uncoated bare substrate and on samples coated on both sides with t_d_ = 40 s and t_d_ = 80 s are reported in Figure 9. Usually, the higher the corrosion potential is, the more difficult the metal oxidation reaction will occur, while the higher the corrosion current density (I_corr_) is, the higher is the corrosion rate and, therefore, the lower is the corrosion resistance.

The values of the corrosion potential E_corr_, obtained with an experimental uncertainty of ±15%, are in good agreement with the range of values reported in the literature for AA2024 alloy samples, 0.6–0.75 V [28], and show slight variations related to the variability of the composition and the test conditions (concentration of electrolyte, temperature, pH). The potential values of the coated samples show an increase in the corrosion potential with respect to the uncoated disks, indicating a greater resistance to corrosion. On the other hand, the current density values show a reduction, indicative of a lower corrosion rate. In order to quantify the effect of TiO_2_ coatings in terms of corrosion resistance, the protection efficiency PE parameter was defined according to the following expression:

(3) $$PE=100*\frac{\;\left(I^{0}\;–\;I\right)}{I^{0}}$$

where *I*^0^ and *I* are the current density values obtained from the potentiometric curves for the bare substrate and the coated samples, respectively. *PE* quantifies the decrease in the corrosion rate of the coated substrates compared to the bare substrate.

The polarization tests that were carried out are summarized in Table 2. For the samples covered on both sides, the results show that as the deposition time increases and, therefore, as the thickness of nano- TiO_2_ on aluminium surface increases, the potential values shift towards nobler potentials. On the other hand, the electric current density is lower on coated samples with respect to the bare substrate, indicating a good resistance to corrosion and a lower corrosion rate. Specifically, *PE* is equal to 75–79% for the sample coated on one side with a deposition time t_d_ = 40 s. *PE* raises up to 87–89% for the sample coated on both sides with the same deposition time. The highest PE is measured to be 90–91% for the samples coated on both sides with an increased deposition time t_d_ = 80 s, and so characterized by a higher thickness of the coating. Those performances are in good agreement with previous works [28], where AA2024 surfaces covered with sol-gel made titania reduced the corrosion current values from 10^−5^ A/cm^2^ down to about 10^−7^ A/cm^2^, with a protection efficiency of about 97%.

**Figure 10:**
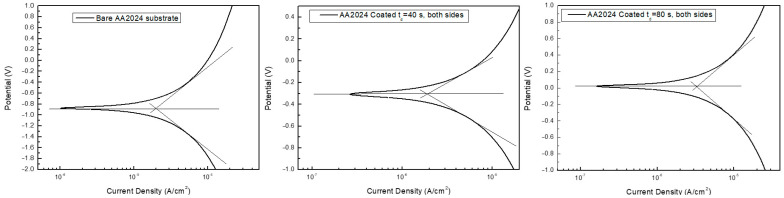
Numerical polarization curves on uncoated bare substrate and on samples coated on both sides with t_d_ = 40 s and t_d_ = 80 s.

The simulations carried out with the COMSOL software are in good agreement with the trend derived from the experimental tests. Particularly, the effect of the coating on both increasing the corrosion resistance compared to the alloy and decreasing the corrosion current was satisfactorily reproduced. However, the model provides the results with some degree of approximation due to several possible causes. The corrosion behavior of the alloy is influenced by the presence of intermetallic phases that act as a cathode or anodic centers, which the model does not take into account. Moreover, the influence of the thickness of titania on the corrosion potential was evaluated, introducing a parameter of porosity of the thickness of titania, which assumes that the coating and the compactness of the coating are expected to increase as the deposition time increases, thus isolating more and more alloy from the electrolyte. Moreover, it is worth pointing out that the model only simulates the behavior of the electrode in a galvanic cell. Typical COMSOL numerical polarization curves are reported in Figure 10, and the results of the simulations are summarized in Table 3.

## 4. Conclusions

This paper presented a technology based on a one-step method for the coating of aluminium alloy surfaces with nanostructured coating layers of TiO_2_ using a combination of aerosol flame synthesis and direct thermophoretic deposition. Nanocoatings of different thicknesses made by 3.2 nm of pure anatase nanoparticles were obtained by varying the total deposition time. A post-deposition thermal annealing treatment was developed. The operating parameters of the thermal annealing treatment were analyzed to obtain an increase in the film crystallization and densification, and so a better adhesion of the coatings to the substrates. UV-vis absorption spectroscopy was used to fine-tuning the annealing treatment. UV-vis spectra were measured on as-deposited coatings and annealed coatings at 300 °C for different times, showing that the thermal treatment does not result in any modification of the optical and structural properties of the nanocoatings.

The morphology of nanoparticles and coatings were analyzed using SEM and TEM microscopy, revealing the presence of uniform coatings of titania nanoparticles on the substrates, as well as a good agreement between the dimension of primary particles in the aggregate and previous dimensional characterizations. The crystalline phase of TiO_2_ was confirmed to be pure anatase, both as nanocoatings and nanopowders, by Raman spectroscopy. The XRD spectra of the coated samples were found to be very similar to the spectra of bare AA2024 samples before the corrosion characterization tests.

Electrochemical polarization measurements were performed to evaluate the corrosion resistance of coated substrates. The electrochemical polarization curves showed a significant increase in the corrosion potential of coated substrates with respect to the bare aluminium and a decrease in the current density. A modeling activity was successfully conducted to confirm and deepen the experimental results. The protection from corrosion of the coatings increased as the deposition time increased, resulting in a greater thickness of the nanolayers. Moreover, a greater resistance to corrosion was obtained by increasing the amount of sample surface protected by nano-titania, performing the coatings procedure on both surfaces of the samples. The best performances in terms of corrosion resistance were obtained for AA2024 substrates coated on both sides with a deposition time t_d_ = 80 s, which showed a protection efficiency higher than 90%. The ability of nano-titania coatings to improve the electrochemical behavior of the aluminum surfaces is likely related to the photocatalytic activity of nano-TiO_2_ and the reductive energy generated under UV irradiation [29,30]. Future works will be devoted to gain a deeper understanding of the corrosion protection mechanisms of nano-TiO_2_ coatings.

## Figures and Tables

**Table 1:** Results of XRD characterization of uncoated and coated disk sample before and after the corrosion test. See also [27] for Al assignment.

2θ	AA2024	AA2024 Corroded	Intensity Ratio	Structural
	Intensity	Intensity		
40.2	2.7	3.16	0.85	Al (111)
46.4	20.11	5.37	3.74	Al (200)
66.5	6.2	1.57	3.95	Al (220)
79.4	19.39	5.7	3.40	Al (331)
84.0	100	100	1	Al (222)
40.2	5.3	5.3	1	Al (111)
46.0	100	100	1	Al (200)
66.5	1.2	1.1	1.1	Al (220)
79.6	1.9	1.9	1	Al (331)
83.4	92.2	91.8	1	Al (222)

**Table 2:** Experimental values of corrosion potential E_corr_, current density I_corr_ and protection efficiency PE from the corrosion tests.

Sample	E_corr_ (V)	E_corr_ (V) ± SD	I_corr_ (A/cm^2^)	PE (%)
**Bare AA2024**	−0.696	−0.67 ÷ −0.71	6.10 × 10^−5^ ÷ 8.40 × 10^−5^	0
**Coated t_d_ = 40 s, one side**	−0.678	−0.63 ÷ −0.69	1.60 × 10^−5^ ÷ 1.90 × 10^−5^	75 ÷ 79
**Coated t_d_ = 40 s, both sides**	−0.275	−0.25 ÷ −0.40	8.20 × 10^−6^ ÷ 9.91 × 10^−6^	87 ÷ 89
**Coated t_d_ = 80 s, both sides**	0.014	0.10 ÷ −0.30	6.62 × 10^−6^ ÷ 8.01 × 10^−6^	90 ÷ 91

**Table 3:** Numerical values of corrosion potential E_corr_, current density I_corr_ and protection efficiency PE from COMSOL simulations.

Sample	E_corr_ (V)	I_corr_ (A/cm^2^)	PE (%) *
**Bare AA2024**	−0.88	6.4 × 10^−5^	0
**Coated t_d_ = 40 s, both sides**	−0.3	5.64 × 10^−6^	92
**Coated t_d_ = 80 s, both sides**	0.024	3.36 × 10^−6^	96

* The values of PE for the coated samples were obtained introducing a porosity parameter.

## References

[1] Starke E.A., Staley J.T. (1996). Application of modern aluminum alloys to aircraft. Prog. Aerosp. Sci..

[2] Williams J.C., Starke E.A. (2003). Progress in structural materials for aerospace systems. Acta Mater..

[3] Davis J.R. (1999). Corrosion of Aluminum and Aluminum Alloys.

[4] Abdeen D.H., El Hachach M., Koc M., Atieh M.A. (2019). A Review on the Corrosion Behaviour of Nanocoatings on Metallic Substrates. Materials.

[5] Liu T., Zhang F., Xue C., Li L., Yin Y. (2010). Structure stability and corrosion resistance of nano-TiO_2_ coatings on aluminum in seawater by a vacuum dip-coating method. Surf. Coat. Technol..

[6] McCracken C., Dutta P.K., Waldman W.J. (2016). Critical assessment of toxicological effects of ingested nanoparticles. Environ. Sci. Nano.

[7] Liberini M., De Falco G., Scherillo F., Astarita A., Commodo M., Minutolo P., D’Anna A., Squillace A. (2016). Nano-TiO_2_ coatings on aluminum surfaces by aerosol flame synthesis. Thin Solid Films.

[8] De Falco G., Porta A., Petrone A., Del Gaudio P., El Hassanin A., Commodo M., Minutolo P., Squillace A., D’Anna A. (2017). Antimicrobial activity of flame-synthesized nano-TiO_2_ coatings. Environ. Sci. Nano.

[9] De Falco G., Ciardiello R., Commodo M., Del Gaudio P., Minutolo P., Porta A., D’Anna A. (2018). TiO_2_ nanoparticle coatings with advanced antibacterial and hydrophilic properties prepared by flame aerosol synthesis and thermophoretic deposition. Surf. Coat. Technol..

[10] Ferreira M.G.S., Zheludkevich M.L., Tedim J., Yasakau K.A. (2012). Self-healing nanocoatings for corrosion control. Corrosion Protection and Control Using Nanomaterials.

[11] Keshavarz M., Idris M.H., Ahmad N. (2013). Mechanical properties of stabilized zirconia nanocrystalline EB-PVD coating evaluated by micro and nano indentation. J. Adv. Ceram..

[12] Li S., Ren Y., Biswas P., Tse S.D. (2016). Flame aerosol synthesis of nanostructured materials and functional devices: Processing, modelling, and diagnostics. Prog. Energy Combust. Sci..

[13] Roth P. (2007). Particle synthesis in flames. Proc. Combust. Inst..

[14] Strobel R., Pratsinis S.E. (2007). Flame aerosol synthesis of smart nanostructured materials. J. Mater. Chem..

[15] Teoh W.Y. (2013). A Perspective on the Flame Spray Synthesis of Photocatalyst Nanoparticles. Materials.

[16] Madler L., Roessler A., Pratsinis S.E., Sahmb T., Gurlo A., Barsan N., Weimar U. (2006). Direct formation of highly porous gas-sensing films by in situ thermophoretic deposition of flame-made Pt/SnO_2_ nanoparticles. Sens. Actuators B.

[17] Tricoli A., Elmøe T.D. (2012). Flame spray pyrolysis synthesis and aerosol deposition of nanoparticle films. AIChE J..

[18] Thimsen E., Rastgar N., Biswas P. (2008). Nanostructured TiO_2_ Films with Controlled Morphology Synthesized in a Single Step Process:  Performance of Dye-Sensitized Solar Cells and Photo Water splitting. J. Phys. Chem. C.

[19] Tolmachoff E., Memarzadeh S., Wang H. (2011). Nanoporous Titania Gas Sensing Films Prepared in a Premixed Stagnation Flame. J. Phys. Chem. C.

[20] Nikraz S., Phares D.J., Wang H. (2012). Mesoporous titania films prepared by flame stabilized on a rotating surface—Application in dye sensitized solar cells. J. Phys. Chem. C.

[21] De Falco G., Commodo M., Minutolo P., D’Anna A. (2019). Flame aerosol synthesis and thermophoretic deposition of superhydrophilic TiO_2_ nanoparticle coatings. Chem. Eng. Trans..

[22] Mahdi R.I., Gan W.C., Abd Majid W.H. (2014). Hot plate annealing at a low temperature of a thin ferroelectric P(VDF-TrFE) film with an improved crystalline structure for sensors and actuators. Sensors.

[23] Martin N., Rousselot C., Rondot D., Palmino F., Mercierc R. (1997). Microstructure modification of amorphous titanium oxide thin films during annealing treatment. Thin Solid Films.

[24] McCormick J.R., Zhao B., Rykov S.A., Wang H., Chen J.G. (2004). Thermal Stability of Flame-Synthesized Anatase TiO_2_ Nanoparticles. J. Phys. Chem. B.

[25] Abdulraheem Y.M., Ghoraishi S., Arockia-Thai L., Zachariah S.K., Ghannam M. (2013). The effect of annealing on the structural and optical properties of titanium dioxide films deposited by electron beam assisted PVD Yaser. Adv. Mater. Sci. Eng..

[26] Luttrell T., Halpegamage S., Tao J., Kramer A., Sutter E., Batzill M. (2014). Why is anatase a better photocatalyst than rutile?—Model studies on epitaxial TiO_2_ films. Sci. Rep..

[27] Wyckoff R.W. (1963). Crystal Structures.

[28] Atta N.F., Abd El Fatah M.A., Galal A. (2017). Effect of titania nanoparticles loading in sol-gel films for corrosion protection of aluminum AA2024-T3 alloy in 3.5% sodium chloride solution. Int. J. Electrochem. Sci..

[29] Ammar S., Cheng C.H., Ma I.A.W., Baig S.B., Kasi R., Subramaniam R., Balakrishnan V. (2020). Effects of TiO_2_ Nanoparticles on the Overall Performance and Corrosion Protection Ability of Neat Epoxy and PDMS Modified Epoxy Coating Systems. Front. Mater..

[30] Tatsuma T., Saitoh S., Ohko Y., Fujishima A. (2001). TiO_2_-WO_3_ Photoelectrochemical Anticorrosion System with an Energy Storage Ability. Chem. Mater..

